# Prevalence and Characteristics of Subjective Cognitive Decline Among Caregivers, 2015-2019

**DOI:** 10.1093/geroni/igab046.1252

**Published:** 2021-12-17

**Authors:** Eva Jeffers, Roshni Patel, Erin Bouldin, Kenneth Knapp, Dana Guglielmo, Christopher Taylor, Lisa McGuire

**Affiliations:** 1 Centers for Disease Control and Prevention (CDC), Atlanta, Georgia, United States; 2 Center for Disease Control and Prevention, Atlanta, Georgia, United States; 3 Appalachian State University, Boone, North Carolina, United States; 4 New York Medical College, Valhalla, New York, United States; 5 CDC, Atlanta, Georgia, United States

## Abstract

Approximately 20% of U.S. adults provide unpaid care to family members and friends with a health condition or disability, and 20% of caregivers reported being in fair or poor health themselves. Much of the assistance caregivers provide have cognitive components, such as medication or financial management, yet little is known about caregivers’ cognitive functioning. Subjective cognitive decline (SCD), or self-reported worsening of memory over the past year, among caregivers could impact the quality of care they provide. This study assessed prevalence of SCD by caregiving status and, among caregivers, the distribution of sociodemographic and other characteristics by SCD status. The study included 93,851 community-dwelling adults aged ≥45 years in 22 states who completed both the Behavioral Risk Factor Surveillance System Cognitive Decline and Caregiving modules during 2015-2019. All data were weighted; comparisons are based on modified Rao-Scott chi-square tests (α=0.05). Among caregivers (n=21,238), 12.6% (95%CI:11.7-13.5) reported SCD, compared with 10.2% (95%CI:9.7-10.7) of non-caregivers (p<0.0001). Caregivers with SCD had more chronic health conditions, lower educational attainment, and were less likely to be married or employed than caregivers without SCD, despite a similar age distribution. Caregivers with SCD were also more likely than caregivers without SCD to report fair or poor health, frequent mental distress, a history of depression, and frequent activity limitations. SCD may negatively impact caregivers’ health, function, and ability to provide care. With the anticipated increases in the need for caregiving, it is critical to understand the cognitive health of caregivers to better support caregivers and care recipients.

